# Alveolar Nitric Oxide as a Biomarker of COVID-19 Lung Sequelae: A Pivotal Study

**DOI:** 10.3390/antiox10091350

**Published:** 2021-08-25

**Authors:** Paolo Cameli, Elena Bargagli, Laura Bergantini, Miriana d’Alessandro, Bruna Giugno, Francesco Gentili, Piersante Sestini

**Affiliations:** 1 Respiratory Diseases Unit, Department of Medical Sciences, Siena University Hospital, 53100 Siena, Italy; bargagli2@unisi.it (E.B.); bergantini@student.unisi.it (L.B.); miriana.dalessand@student.unisi.it (M.d.); brunamaria.giugno@student.unisi.it (B.G.); sestini@unisi.it (P.S.); 2 Unit of Diagnostic Imaging, University Hospital Santa Maria alle Scotte, 53100 Siena, Italy; francesco.gentili@ao-siena.toscana.it

**Keywords:** nitric oxide, COVID-19, biomarkers, SARS-CoV-2

## Abstract

Since SARS-CoV-2 emerged in 2019, strict monitoring of post-COVID-19 patients in order to ensure the early detection of sequelae and/or chronic organ damage that could been associated with the infection has been essential. Potential involvement of the NO pathway in the development of post-COVID-19 lung fibrotic alterations is feasible, since the majority of respiratory cells can produce NO, and fractional exhaled NO (FeNO) represents a biomarker of airway inflammation. The aim of this study was to investigate the potential utility of multiple-flow FeNO parameters in a post-COVID-19 population and to compare it with other indicators of lung damage proposed in the literature. We enrolled 20 patients hospitalized for COVID-19, who underwent clinical, respiratory functional (including PFTs and FeNO) and radiological follow-up after discharge. Compared with age- and sex-matched healthy controls, post-COVID-19 patients showed significantly higher FeNO 350 mL/s and CaNO levels. Moreover, among the parameters included in the follow-up, CaNO showed the best accuracy in indicating predominant fibrotic changes and GGO at CT scan. To our knowledge, this preliminary study has investigated for the first time multiple-flow FeNO parameters in a post-COVID-19 population. The evidence of increased CaNO values may imply the persistence of alveolar and bronchiolar inflammation and/or a mild impairment of the alveolar-capillary membrane in these patients.

## 1. Introduction

Since December 2019, severe acute respiratory syndrome coronavirus-2 (SARS-CoV-2)-induced infection has spread globally, reaching the status of pandemic in March 2020, as declared by the World Health Organization [1]. The SARS-CoV-2 pandemic is ongoing and still represents a global health menace, due to its high transmissibility and the risk of inducing severe respiratory failure, caused, for example, by acute respiratory distress syndrome (ARDS). This has severely hindered national health systems over the last 18 months [2].

Vaccines and specific, although still unsatisfactory, therapeutic approaches have been shown to reduce mortality, hospitalizations, and the risk of invasive mechanical ventilation due to coronavirus-19 disease (COVID-19) [3,4,5,6]. However, regardless of the severity of the primary disease, there are many patients who may develop long-term symptoms after COVID-19 that significantly impair quality of life, and for whom no adequate treatment or preventive measures are available [7,8]. Moreover, since SARS-CoV-2 emerged in 2019, it has become essential to strictly monitor post-COVID-19 patients to ensure early detection of long-term sequelae and chronic organ damage associated with the infection.

Among these sequelae, in the case of severe pulmonary disease, lung fibrosis may be considered as one of the most dangerous and expected complications of COVID-19. This assumption is further strengthened by two aspects: first, COVID-19 may evolve into a condition similar to ARDS, which in turn is known to induce irreversible pulmonary fibrosis in a relevant percentage of patients, as a consequence of an aberrant and dysregulated repair processes during the fibrotic phase of disease [9,10]. Second, viral infections and the associated cytopathic effects on the alveolar epithelium can trigger early pathogenic processes, leading to the onset of idiopathic pulmonary fibrosis (IPF) [11]. Preliminary evidence suggests that SARS-CoV-2 may induce lung fibrogenesis through different pathways [12,13,14], but the clinical relevance of these processes is as yet unclear. CT scans can show typical parenchymal fibrotic strands in post-COVID-19 patients, as in other diffuse viral pneumonias (such as H1N1-influenza [15]), and in a certain percentage of patients, a diffuse and apparently progressive interstitial pneumonia may be observed [16,17]. However, to date, the potentially progressive nature of these fibrotic changes caused by COVID-19 remains a matter of debate, as is their impact on respiratory physiology; in the same way, few biomarkers have been investigated so far to assess their relationship with fibrotic sequelae [18].

Among exhaled biomarkers, exhaled nitric oxide (NO) is by far one of the most studied and validated in clinical practice, especially for the management of asthma. The majority of respiratory cells can produce NO, and FeNO represents a reliable non-invasive biomarker of airway inflammation [19]. Recent studies have demonstrated that NO plays a crucial role as an intra- and extracellular mediator in asthma, but also in lung fibrogenesis [20]. In fact, murine and human models of lung fibrosis revealed increased production of NO by iNOS, suggesting the potential role of NO not only in perpetuating, but also in triggering the onset of interstitial lung involvement [21,22]. From these assumptions, the potential involvement of the NO pathway in the development of lung fibrotic alterations post-COVID-19 is feasible. The aim of this study was to investigate multiple-flow FeNO parameters in a post-COVID-19 population to evaluate the potential persistence of alveolar and bronchiolar inflammation in these patients.

## 2. Materials and Methods

### 2.1. Study Population and Study Design

This observational study was performed at the Siena Regional Referral Center for Interstitial Lung Diseases, Siena University Hospital, from July 2020 to September 2020. The study population was composed of 20 post-COVID-19 patients (13 males, 60 ± 14.3 years old) hospitalized in the COVID Unit of Siena University Hospital for COVID-19. All these patients had agreed to undergo to the local follow-up protocol for the evaluation of post-COVID-19 assessment. Exclusion criteria were:

- Inability or refusal to provide informed consent
- Comorbidities that may influence FeNO values: asthma, allergic diseases, bronchiectasis, cystic fibrosis, pulmonary arterial hypertension, pre-existent interstitial lung disease, chronic rhinosinusitis, with or without nasal polyps
- Ongoing treatment with oral phosphodiesterase-5 inhibitors, oral or inhaled corticosteroids.

According to our Centre protocol, a follow-up visit was scheduled about three months after hospital discharge. Post-COVID-19 follow-up included a respiratory physician assessment, pulmonary function tests (PFTs), including the evaluation of diffusion lung capacity, chest high resolution computed tomography (HRCT) and multiple-flow analysis of fractional exhaled nitric oxide (FeNO). Demographic and clinical data, including those referring to COVID-19 severity, length of hospital stay and pharmacological treatment during hospitalization, were collected and entered into an electronic database for statistical analysis, along with respiratory functional (FeNO) parameters and radiological data.

Concerning the CT scan, a radiologist experienced in thoracic imaging was asked to detect the predominant feature among: organizing pneumonia (OP), air-trapping, ground glass opacities (GGO) and fibrotic abnormalities. Chest HRCT scans were performed according the scanning parameters recommended by ATS/ERS guidelines for diagnosis of ILD [23].

As a control group, we also included multiple-flow analysis of FeNO from a historic cohort of 22 healthy volunteers (15 males, 58.5 ± 7.4 years old). To be included in the study, healthy controls must not have been taking pharmacological drugs interfering with FeNO measurements, such as phosphodiesterase-five inhibitors and inhaled corticosteroids, in the last three months. All healthy volunteers had a normal lung function test and they did not report respiratory symptoms or infections within the last 4 weeks before the FeNO assessment. To be included in the study, healthy controls were required not to have been infected by SARS-CoV-2.

All patients included in the study gave their written informed consent to participate in the study, which was approved by our Local Ethics Committee (C.E.A.V.S.E. Markerlung 17431). The study was conducted in accordance with the Declaration of Helsinki.

### 2.2. Pulmonary Function Tests

The following lung function measurements were recorded according to ATS/ERS standards [24,25], with corrections for temperature and barometric pressure: forced expiratory volume in the first second (FEV1), forced vital capacity (FVC), FEV1/FVC, total lung capacity (TLC), residual volume (RV), carbon monoxide lung transfer factor (TLCO) and carbon monoxide lung transfer factor/alveolar volume (TLCO/VA). PFTs were performed after FeNO assessment. All measurements were performed using a Jaeger Body Plethysmograph that was calibrated daily.

### 2.3. Multiple-Flow FeNO Assessment and Analysis

Multiple-flow FeNO measurements were performed using an electrochemical analyser (model Hypair FeNO Medisoft Cardioline Exp’air, Medisoft, 5503 Sorinnes, Belgium 2010) according to ATS recommendations for online measurement of FeNO in adults [26]. The analyser was sensitive from 1 to 500 ppb NO with a resolution of 1 ppb. All measurements were made at an ambient NO concentration of <10 ppb. Exhaled NO was measured during slow exhalation from total lung capacity against positive pressure in the range of 5–20 cm H_2_O. Exhalation flow rate was kept constant by a biofeedback visual display. FeNO was measured at flow rates of 50, 100, 150 and 350 mL/s. For each flow rate, at least two technically satisfactory measurements were performed, and in the case of a difference of more than 10% between these measurements, a third measurement was taken. The flow-independent NO parameters, CaNO, and maximum airway flux of NO (J’awNO) were calculated by the device software using the linear model endorsed by the recent ERS technical standard. For each patient, the linear relationship was evaluated between the three points (100, 150 and 350 mL/s) of NO flux versus flow. Each measurement was considered acceptable with a confidence rate > 95% and a flow stability > 90%. All FeNO measurements were performed by a single investigator, experienced in FeNO measurements.

### 2.4. Statistical Analysis

All statistical analyses were performed using parametric or non-parametric tests, according to normality distribution of variables. Normality tests included the Kolmogorov–Smirnov test and the D’Agostino–Pearson test. Comparisons of FeNO levels between patients and controls were made using the Mann–Whitney test and median tests, while correlations between variables were performed using the Spearman test. Demographic features (except for BMI) showed a normal distribution and therefore were compared through *t*-test. Data is expressed as mean ± standard deviation (SD), unless otherwise reported. A cut-off of *p*-value < 0.05 was selected for statistical significance. Statistical analysis, receiving operating characteristic (ROC) curves and figures were conducted with Stata version 15.1 (Stata corp, College Station, TX, USA), Microsoft Excel and Graphpad Prism 5.0 for Windows.

## 3. Results

### 3.1. Study Population

All 20 patients enrolled in the study underwent a follow-up visit 93.4 ± 15 days after hospital discharge (102.9 ± 16.5 days from symptomatic onset of disease). There was no statistical difference in terms of age, sex prevalence or smoking status between patients and healthy controls (*p* > 0.05 for all). Clinical and demographic data, as well as respiratory functional parameters and chest HRCT features are reported in Table 1. Regarding respiratory comorbidities, three patients were affected by chronic obstructive pulmonary disease (COPD) and they were all treated with long-acting muscarinic agents (LAMA). None of the patients included in the study reported a diagnosis of asthma, allergic rhinitis, chronic rhinosinusitis with nasal polyps, bronchiectasis, or underlying interstitial lung diseases, and/or were treated with ICS, oral steroids, or phosphodiesterase-5 inhibitors. No significant differences were observed in terms of functional parameters, CT features and FeNO values between male and female patients.

Concerning COVID severity and pharmacological treatment, 14 patients (70%) (nine males) experienced acute respiratory failure secondary to COVID-19 and 11 (55%) needed mechanical ventilation plus oxygen therapy during hospitalization. Among these, four patients (three males) underwent orotracheal intubation and invasive mechanical ventilation in the COVID Intensive Care Unit (ICU).

Regarding PFTs, we did not observe any statistically significant alterations of lung volumes and/or diffusion lung capacity for carbon monoxide, on average, independently from lung disease severity. Specifically, five patients (25%) showed an impairment of DLCO (four mild and one moderate) and three (15%) patients reported a reduction of FVC (two mild and one moderate); altogether, an obstructive or restrictive defect at PFTs was observed in five (25%) and two patients (10%), respectively.

### 3.2. FeNO Measurements

**Figure 1:**
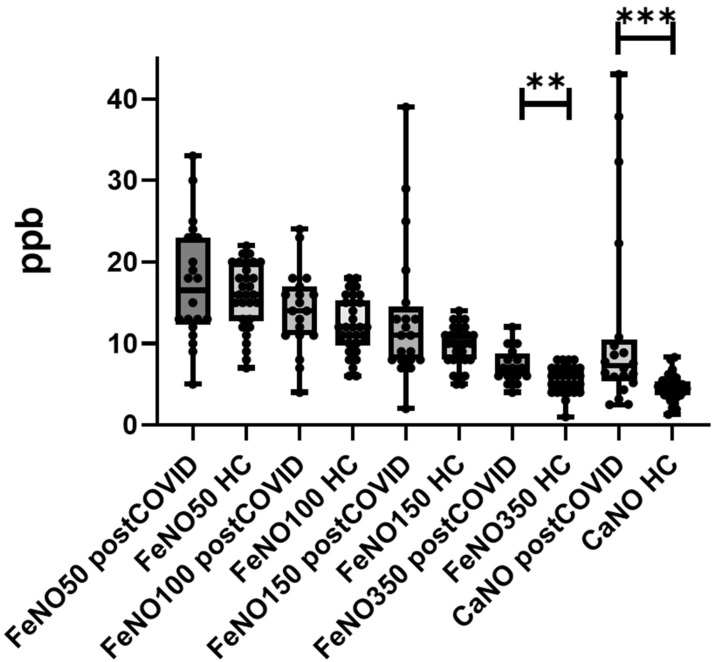
Comparison of multiple-flow FeNO parameters between healthy controls (HC) and post-COVID-19 patients. Ppb: pars per billion; CaNO: alveolar concentration of nitric oxide; FeNO: fractional exhaled nitric oxide; **: *p* = 0.019; ***: *p* = 0.0081.

Multiple-flow FeNO values, as well as flow-independent parameters (CaNO and J’awNO), are reported in Table 2. Post-COVID-19 patients reported significantly higher FeNO 350 mL/s and CaNO levels than healthy controls (*p* = 0.0199 and *p* = 0.0081, respectively) (Figure 1), while no significant differences were found in terms of FeNO 50, 100 and 150 and J’awNO parameters. We did not observe any difference among FeNO data after stratification of the study population according to age > 65 years, COVID-19 severity, need for oxygen supplementation or mechanical ventilation, ICU acceptance, use of steroids and/or remdesivir and/or interleukin-6 inhibitors during hospitalization.

### 3.3. Correlations with Radiological Features

The radiological features of our study population are reported in Table 1. In our population, only 5 patients (25%) did not show any radiological abnormalities in the CT scan. Patients that experienced acute respiratory failure during hospitalization were more likely to develop fibrotic abnormalities at the follow-up CT scan (*p* = 0.0419), regardless of the need for mechanical ventilation and/or high-flow oxygen therapy and/or orotracheal intubation. In the same way, patients with lung fibrotic changes showed a significantly higher length of stay in hospital (*p* = 0.0383), while no differences were observed in terms of ventilator-free days or length of stay in ICU (*p* > 0.05 for both). The presence of air trapping, OP and/or GGO was not associated with any of the clinical outcomes investigated in this study.

**Figure 2:**
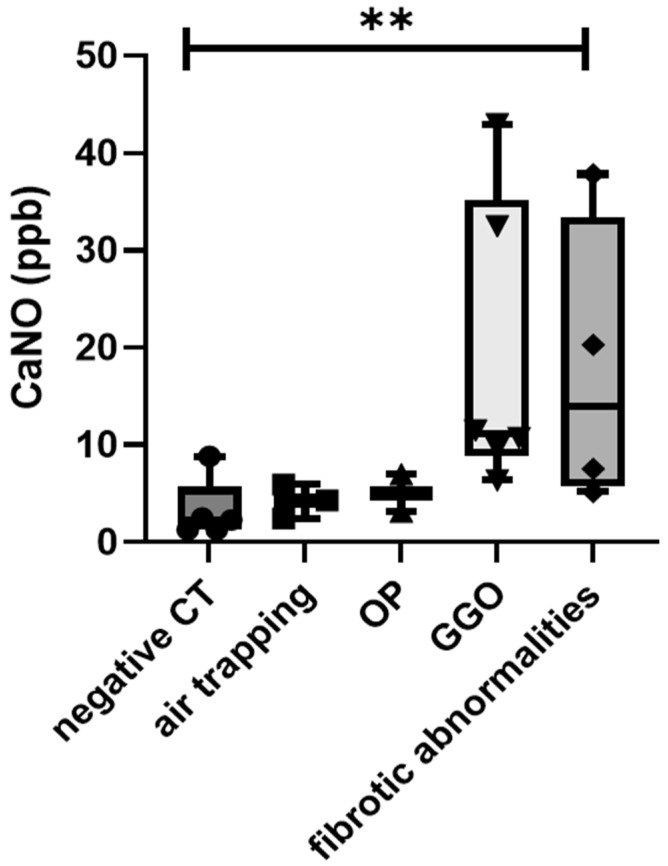
Comparison of CaNO values of post-COVID-19 patients among different CT phenotypes. **: *p* = 0.0291.

Regarding the correlation between CT features and FeNO parameters, only CaNO showed significant differences across the different CT features; in particular, predominant fibrotic changes and GGO were associated with the highest CaNO levels (*p* = 0.0291) (Figure 2). No other significant differences were observed among FeNO parameters and CT scan data.

**Figure 3:**
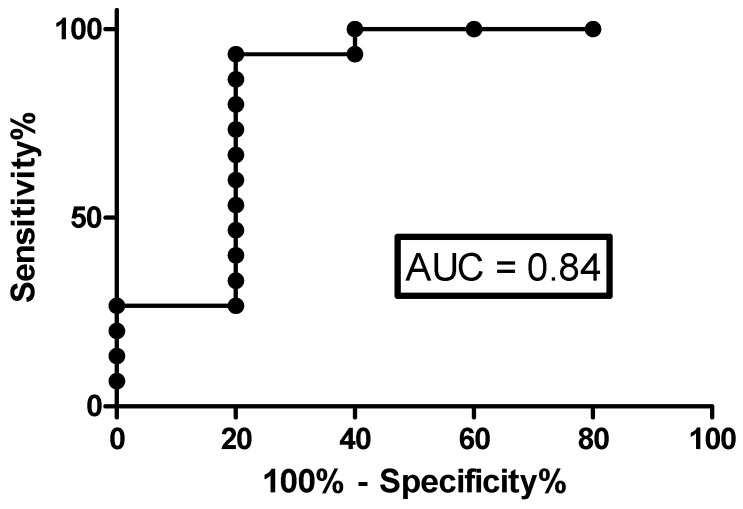
ROC curve for assessment of accuracy of CaNO in detecting CT lung abnormalities.

To investigate the accuracy of the FeNO and respiratory functional parameters (FVC, FEV1, TLC and DLCO) for the detection of CT lung abnormalities, we performed ROC analysis. CaNO showed the best performance among these parameters (AUC 0.84, *p* = 0.02607; sensitivity 73.3% and specificity 80%, for a cut-off value of 5.5 ppb, positive predictive value 0.89, likelihood ratio 4.33) (Figure 3).

We did not observe any significant differences in respiratory functional parameters, including DLCO, among patients with different radiological CT features and/or different clinical outcomes due to COVID-19. Only two patients (10%), of whom one reported a diagnosis of COPD, showed a mild to moderate reduction in lung volumes combined with a moderate impairment of DLCO. ROC analysis performed for the accuracy of FVC, FEV1, TLC and DLCO in the detection of lung CT abnormalities were all insignificant (AUC 0.57, *p* = 0.6312; AUC 0.53, *p* = 0.8273; AUC 0.75, *p* = 0.0956 and AUC 0.52, *p* = 0.8958, respectively).

## 4. Discussion

In this study, we reported our preliminary experience of a multidimensional follow-up evaluation of early post-COVID-19 patients. All our patients had been hospitalized due to COVID-19 and the majority of them needed oxygen therapy as support treatment during hospitalization. Despite the high percentage of severe or critical cases, we did not observe a significant impairment of lung volumes and diffusion lung capacity in our population, suggesting that COVID-19 may not necessarily induce irreversible lung damage in terms of respiratory functions. On the other hand, most of our patients reported persistent lung abnormalities at CT scan, including specific radiological features such as air trapping, GGO, fibrotic alteration and OP, that typically involve different areas of lung architecture: since 17/20 patients did not report any respiratory disorder in their medical history, our data was of interest, suggesting that COVID-19 may induce small airway disease in addition to long standing GGO and fibrotic strands. The radiological features were not associated with a significant impairment of gas exchange or lung volumes; however, it was confirmed that the persistence of air-trapping after COVID-19 contributes to the persistent respiratory symptoms of post-COVID-19 syndrome, as already suggested in the literature [27].

Interestingly, we also observed significantly higher values of CaNO and distal FeNO (350 mL/s) in post-COVID-19 patients than in healthy sex- and-age matched controls. These findings are somewhat expected, since NO is a crucial mediator in the amplification and modulation of inflammatory tone of the airways and may also be produced through the inducible NO synthase (iNOS) expressed by alveolar macrophages and pneumocytes [21,28,29]. Indeed, increased distal FeNO and CaNO values have been reported in different parenchymal lung disorders, including fibrotic interstitial lung diseases or inflammatory disorders, while no specific data are available on viral pneumonia [30,31].

To our knowledge, this preliminary study has investigated for the first time, multiple-flow FeNO parameters in a post-COVID-19 population. The evidence of increased CaNO values may imply the persistence of alveolar and bronchiolar inflammation and/or mild impairment of the alveolar-capillary membrane in these patients. Notably, CaNO values were significantly higher in patients with GGO and fibrotic alterations at CT scan at the follow-up, showing a better accuracy in the detection of these lung abnormalities with respect to other PFT parameters, including DLCO. Considering that our population reported no specific alterations in lung volumes or DLCO, these results suggest that CaNO may be more sensitive than conventional functional parameters in detecting interstitial/alveolar lung damage and, therefore, could be useful in the follow-up of those patients who experience persistent respiratory symptoms, despite normal PFTs. Compared with the available literature [32,33,34,35], in our population DLCO impairment was observed in a minority of patients and appeared not to be associated with CT lung alterations. This could be related to the small sample size of the study and to referral bias, since patients still needing oxygen therapy or unfit to undergo follow-up evaluation were not included in the study. However, lung diffusion for nitric oxide has already demonstrated better sensitivity than DLCO in detecting alveolar impairment in ILD patients [36,37]. These assumptions may explain the better performance of CaNO with respect to DLCO in our study. These results are certainly intriguing, since they support the potential role of CaNO as a biomarker of lung inflammation in post-COVID-19 patients and may suggest interesting insights into the pathogenesis of pulmonary fibrotic sequelae involving the NO pathway.

This research has some limitations: first, the limited sample size and the monocentric nature of the study; secondly, none of our patients had ever undergone a CT scan, PFT or FeNO measurements before the present study and, therefore, we were unable to compare our findings with pre-COVID-19 test results. Therefore, despite the detailed collection of medical history, we cannot exclude pre-existing medical conditions that may have influenced radiological features and respiratory functional parameters, leading to a higher risk of collection bias.

## 5. Conclusions

In conclusion, our study reported increased CaNO and distal FeNO values in post-COVID-19 patients, supporting the correlation of pulmonary long-lasting inflammation status in these subjects with the persistence of GGO or the development of fibrotic lung alterations. Measurement of CaNO levels demonstrated the best accuracy in the detection of radiological sequelae of COVID-19, and this may be suggested as a non-invasive biomarker to be implemented in the follow-up of these patients, given its reproducibility and non-invasive procedure.

## Figures and Tables

**Table 1:** Demographic, functional and predominant radiological features of COVID-19 patient cohort.

Parameters	Post-COVID	Healthy Controls	*p*-Value
N°	20	22	
Age (years)	60 ± 14.3	58.5 ± 7.4	0.6648
Male (%)	13 (65)	15 (68.2)	0.9851
Smoking status (pack/year)	10.6 ± 5.4	8.4 ± 6.6	0.2567
BMI (kg/m^2^)	24.3 ± 5.4	23.8 ± 4.3	0.5598
Time from onset to hospitalization (d)	8.5 ± 4.3		
Length hospital stay (d)	16.7 ± 14.2		
Acute respiratory failure (%)	14 (70)		
ICU admittance (%)	4 (20)		
*Therapy*			
Steroid (%)	14 (70)		
Remdesivir (%)	5 (25)		
Anti IL-6 drugs (%)	6 (30)		
*PFTs*			
FVC l (%)	3.6 ± 1.2 (99 ± 21)		
FEV1 l (%)	2.7 ± 0.9 (92.8 ± 17.2)		
FEV1/FVC	76.2 ± 9.2		
RV l (%)	2.2 ± 0.6 (99.6 ± 19.8)		
TLC l (%)	5.9 ±1.2 (95.5 ± 15)		
DLCO %	94 ± 19.3		
KCO %	106.5 ± 21.5		
*CT features*			
Negative (%)	5 (25)		
Air trapping (%)	3 (15)		
OP (%)	2 (10)		
GGO (%)	6 (30)		
Fibrotic abnormalities (%)	4 (20)		

DLCO: diffusing lung capacity for carbon monoxide; FEV1: Forced Expiratory Volume in the 1st second; FVC: Forced vital capacity.

**Table 2:** Multiple-flow FENO parameter values between post-COVID-19 and HC. *: Mann–Whitney U test; °: median test.

Multiple-Flows FeNOParameters	Post-COVID	Healthy Controls	*p*-Value
FeNO 50 (ppb)	17.3 ± 7.2	15.8 ± 4.1	0.6336 *
Min-max; 25–75 percentile (ppb)	5–33; 12.2–23	7–22; 12.7–20	Chi-square 0.53, *p* = 0.817 °
FeNO 100 (ppb)	14.1 ± 5.1	12.2 ± 3.5	0.1675 *
Min-max; 25–75 percentile (ppb)	4–24; 11–17	6–18; 9.7–15.2	Chi-square 2.49, *p* = 0.114 °
FeNO 150 (ppb)	13.3 ± 8.7	9.7 ± 2.4	0.2437 *
Min-max; 25–75 percentile (ppb)	2–39; 8–14.5	5–14; 8–11.2	Chi-square 0.65, *p* = 0.419 °
FeNO 350 (ppb)	7.2 ± 2.1	5.6 ± 1.6	0.0199 *
Min-max; 25–75 percentile (ppb)	4–12; 6–8.7	1–8; 4–7	Chi-square 4.18, *p* = 0.041 °
J’awNO (nL/min)	44.1 ± 31.9	45.3 ± 22.3	0.6936 *
Min-max; 25–75 percentile (ppb)	2.8–129.8; 18.6–57.4	5.6–90.4; 28.5–54.9	Chi-square 0.35, *p* = 0.551 °
CaNO (ppb)	9.7 ± 6.1	4.5 ± 1.6	0.0081 *
Min-max; 25–75 percentile (ppb)	1.3–43.1; 5.8–20.6	1.2–8.3; 3.6–5.3	Chi-square 7.57, *p* = 0.006 °

FeNO: fractional exhaled nitric oxide; J’awNO: bronchial maximum flux of NO; CaNO: alveolar concentration of NO.

## Data Availability

The data presented in this study are available in Results’ Section.
